# Garré’s Sclerosing Osteomyelitis of the Tibia in a Hispanic Adult Male: A Case Report

**DOI:** 10.7759/cureus.57837

**Published:** 2024-04-08

**Authors:** Hector Sanchez-Fernandez, Alexandra Claudio-Marcano, Miguel Gonzalez-Ugarte, Norman Ramírez-Lluch, Juan Bibiloni

**Affiliations:** 1 Orthopaedic Surgery, University of Puerto Rico, Medical Sciences Campus, San Juan, PRI; 2 Pediatric Orthopedic Surgery, Mayagüez Medical Center, Mayagüez, USA

**Keywords:** inflammatory marker, bone scan, bone biopsy, midshaft tibia, garre's osteomyelitis

## Abstract

Sclerosing osteomyelitis of Garré is a rare inflammatory pathology characterized by cortical thickening and loss of the medullary canal. Typically, this pathology affects the mandible. However, the involvement of long bones, such as the femur and tibia, is also possible. This condition predominantly affects children and young adults, especially females, and commonly emerges before age 25, with an average onset age of 16 years. The disease is characterized by an insidious onset, causing local pain, distention of the affected bone, and a moderately increased erythrocyte sedimentation rate. We aim to report a unique case involving a 25-year-old Hispanic male presenting with a one-year insidious onset of left anterior lower leg pain. The patient’s clinical course, laboratory findings, and imaging results are discussed. Despite a three-month trial of conservative management, symptomatic relief was elusive, prompting a left tibia core biopsy. Biopsy results revealed an inflammatory-reactive process with a xanthogranulomatous reaction. The continuation of conservative measures post-biopsy led to significant symptom resolution, highlighting the potential efficacy of histopathological examination. This case contributes to the limited literature on adult sclerosing osteomyelitis of Garré, particularly in long bones and among Hispanic individuals. Successful management through biopsy and conservative treatment provides valuable insights into therapeutic options for this rare condition.

## Introduction

Garré sclerosing osteomyelitis is a rare inflammatory disease of chronic nature characterized by thickening of the cortices and loss of the medullary canal [1]. This pathology was first described by the Swiss surgeon Carl Philip Garré in 1893 [2]. It is a well-described entity in the dental literature, and the most commonly involved bone is the mandible [3]. However, the involvement of long bones, such as the femur and tibia, has also been reported in the literature [4]. This condition mainly affects children and young adults, with only a few cases of adults reported in the literature [2,3,5-7]. It commonly occurs before the age of 25 years due to the peak of periosteal osteoblastic activity [8], with a mean onset age of 16 years [9]. Females are more likely to be affected, with a female/male ratio of 5:1 [7]. Such disease usually affects the anterior aspect of the tibia and the lateral surface of the body of the mandible [8]. The clinical course is characterized by an insidious onset with local pain, distention of the affected bone, and a moderately increased erythrocyte sedimentation rate [9]. Additionally, a secondary lesion can occur at a distant site many years after the initial onset [4]. Differential diagnoses can include several conditions, such as osteoid osteoma, osteosarcoma, or Ewing’s sarcoma [7]. To the best of our knowledge, no distinctive diagnostic criteria are discussed in the literature [3]. Thus, sclerosing osteomyelitis of Garré is a diagnosis of exclusion after more aggressive conditions have been ruled out and a thorough workup has been done [3].

Adequate treatment of sclerosing osteomyelitis remains unclear [3], with symptomatic relief being the aim in most cases [9]. Patients have been shown to respond well to analgesics, non-steroidal anti-inflammatory drugs (NSAIDs), or antibiotic therapy, although the exact mechanism remains unclear [4]. Furthermore, a biopsy with the opening of the medullary canal sometimes decreases symptoms [9]. In cases where positive outcomes cannot be achieved with non-invasive treatments, surgical procedures are recommended as a last-option resource [3].

After a thorough literature review, we only found five cases of Garré’s sclerosing osteomyelitis of the tibia in adult patients [2,3,5-7]. Only two of these were males [5,7], and no instances of Hispanics have been reported before. This study aims to report a rare case of sclerosing osteomyelitis of Garré affecting the left anterior tibia midshaft of a 25-year-old adult Hispanic male, whose diagnosis was made by clinical-radiological correlation and biopsy.

## Case presentation

We present the case of a 25-year-old male who complained about a one-year-long left anterior lower leg pain of insidious onset. According to the patient, the pain was exacerbated by exercise. He also complained that it had been worsening since its onset, to the point where he was awoken from sleep and had periods where he was unable to walk due to the severity of the severity of the pain. The pain was temporarily alleviated with over-the-counter NSAID medication, rest, and avoiding exercise. The patient denies fever, weight loss, or superficial edema but states that he had a period of one month where the overlying skin was warm to touch and tender to palpation. On a physical exam, the left lower leg had no overlying edema, erythema, warmth, or tenderness to palpation. The patient had no gait abnormalities and was neurovascularly intact.

**Figure 1:**
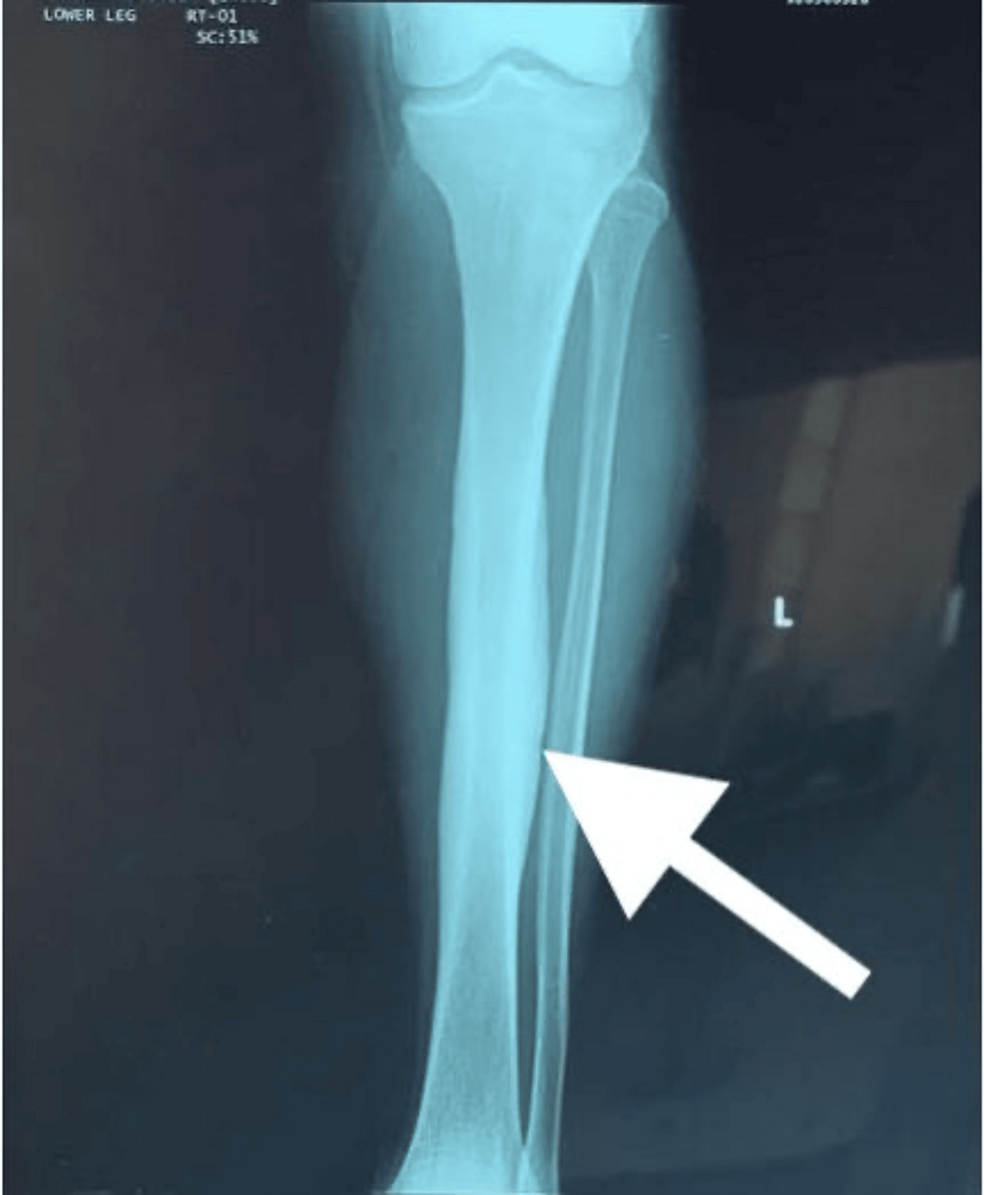
An AP view of the left tibia. Thickening of the cortices of the mid-diaphysis of the tibia can be appreciated.

**Figure 2:**
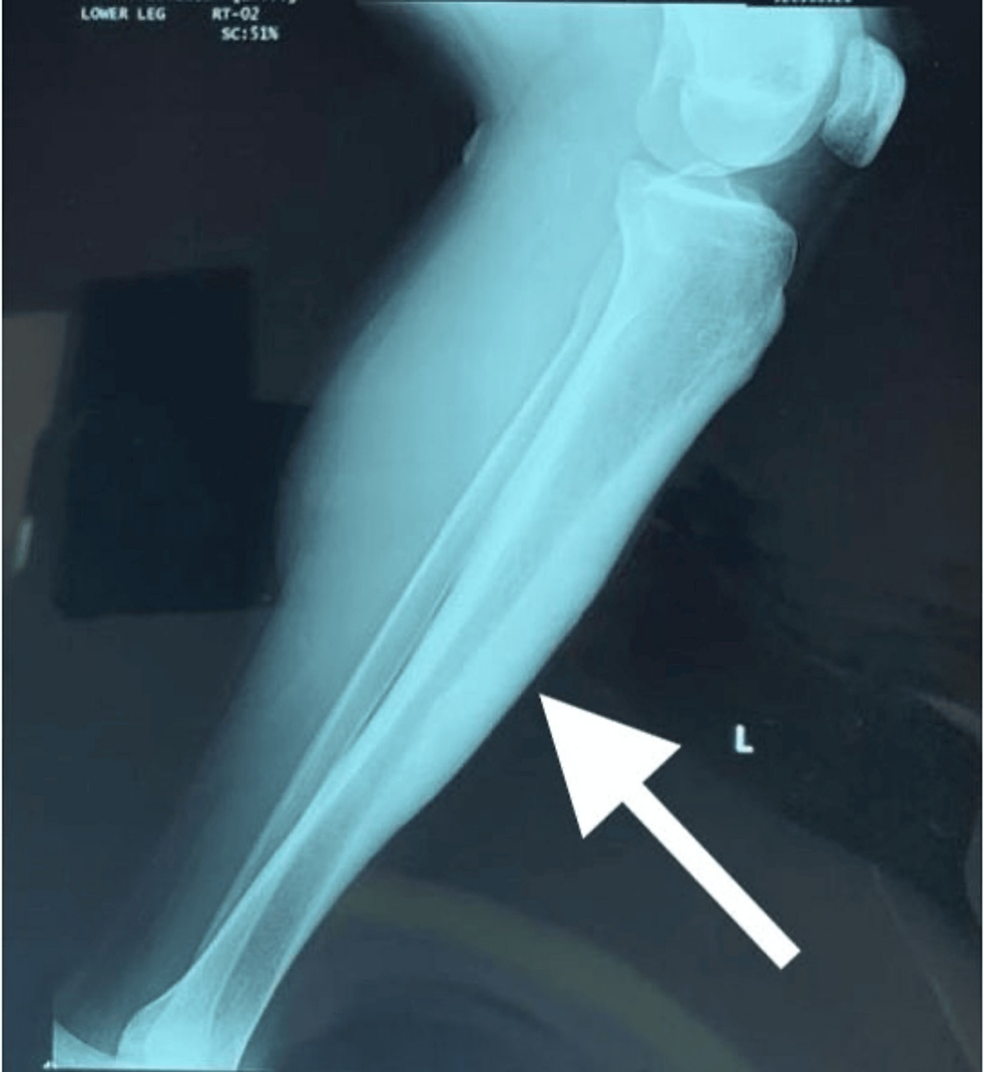
Lateral view of the left tibia. Thickening of the anterior cortex can be appreciated.

**Figure 3:**
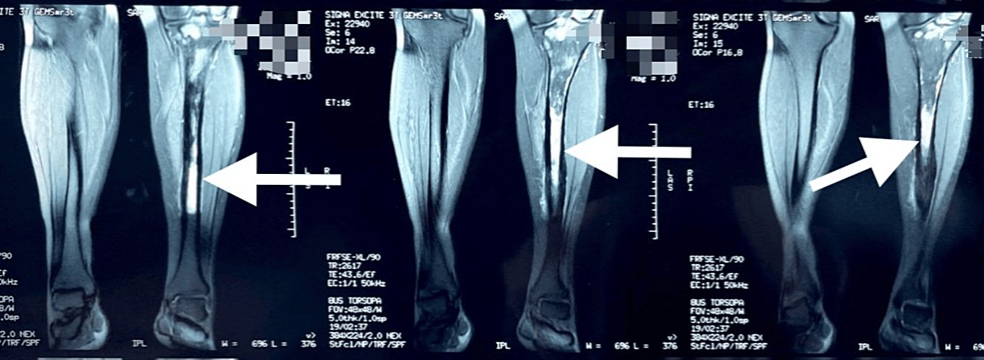
MRI

**Figure 4:**
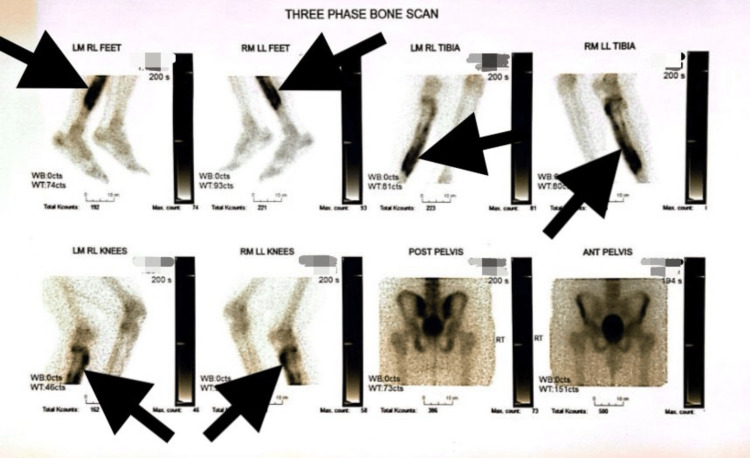
Three-phase bone scan.

The patient’s laboratory results were remarkable for elevated ESR and CRP of 30 mm/hr and 5 mg/dL, respectively. Left lower extremity X-rays demonstrated thickening of the anterior cortex of the tibia and showed no evidence of fracture or fibula abnormalities (Figures 1, 2). Magnetic resonance imaging (MRI) without contrast was remarkable again for cortical thickening in the region of the anterior tibia mid-shaft. Soft tissue was unremarkable for abnormalities or injuries (Figure 3). A three-phase bone scan was then performed and was remarkable for increased radiotracer uptake in the proximal aspect of the left tibia laterally and on the left tibial plateau (Figure 4). There was also diffusely increased focal radiotracer uptake in the left temporal bone, extending into the left temporomandibular joint. Diffusely increased radiotracer uptake was also observed throughout both sacroiliac joints and wings. 

**Figure 5:**
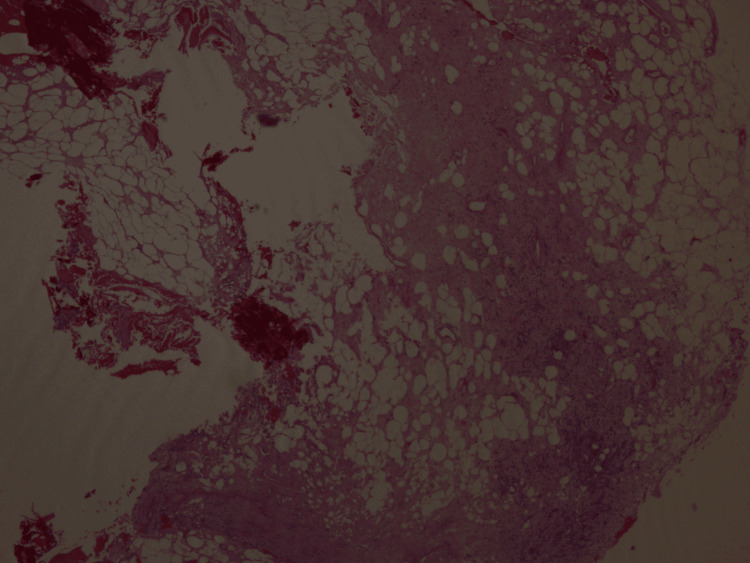
Biopsy pathology results. Inflammatory reactive process of undetermined etiology with xanthogranulomatous reaction.

**Figure 6:**
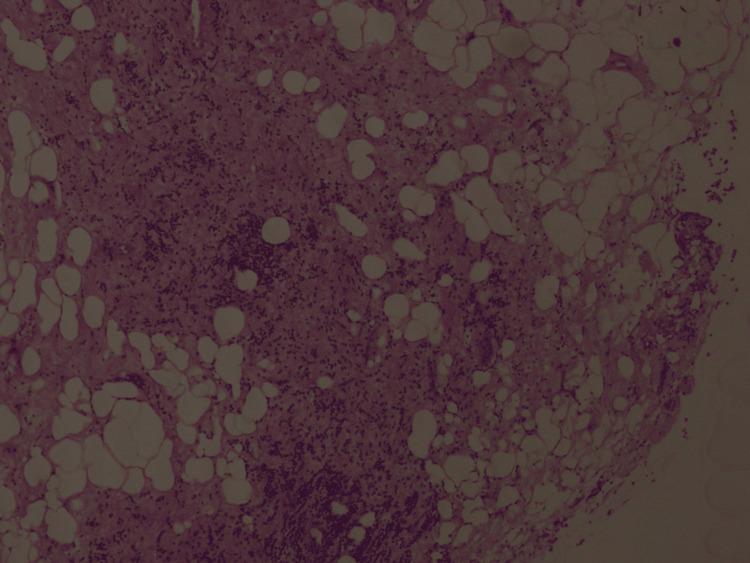
Biopsy pathology results. Inflammatory reactive process of undetermined etiology with xanthogranulomatous reaction.

After a three-month trial of conservative management using Naproxen 500mg PO q12h without symptom relief, a core biopsy of the left tibia was performed to rule out malignant processes such as Ewing’s sarcoma or osteosarcoma. The cultural results came back negative. Pathology results showed an inflammatory-reactive process of undetermined etiology with a xanthogranulomatous reaction (Figures 5, 6). After the biopsy, the patient continued to be managed with Naproxen 500mg PO q12h, with a gradual improvement in symptoms. One year after the biopsy, the patient denied pain, was able to return to his activities of daily living, and expressed that his quality of life had improved drastically. 

## Discussion

Sclerosing osteomyelitis of Garré is a rare pathology with unique characteristics that pose a diagnostic challenge. The chief complaint of patients affected by sclerosing osteomyelitis of Garré is localized pain to the diaphysis of the affected bone and tenderness at the area, with preserved bone functioning [1,2]. The duration of symptoms is very variable and may persist for several months or years [1]. Constitutional symptoms are usually absent [4]. A physical examination tends to be unremarkable [3]. Meanwhile, laboratory results often show elevated levels of inflammatory markers such as erythrocyte sedimentation rate (ESR) and C-reactive protein (CRP) [4]. Radiographic imaging commonly presents with reduced medullary canals and stiffened cortices [10]. Isotope scans may show increased uptake at the involved site [1]. Additionally, biopsy specimens show only chronic, low-grade, non-specific osteomyelitis, and cultures are usually negative [9].

In this case, the patient presented after a one-year-long insidious onset of left anterior lower leg pain that had been worsening since its development. He stated that the pain increased in severity with exercise and awoke him from sleep. His physical examination was unremarkable. Additionally, his ESR and CRP were elevated, and imaging studies were remarkable for cortical thickening in the region of the anterior tibia mid-shaft. Lastly, his biopsy results showed an inflammatory-reactive process of undetermined etiology with a xanthogranulomatous reaction.

Distinguishing sclerosing osteomyelitis of Garré from other bone pathologies such as stress fracture, osteoid osteoma, osteosarcoma, or Ewing’s sarcoma is challenging, often making it a diagnosis of exclusion after other aggressive conditions are ruled out. Lack of distinctive diagnostic criteria requires a comprehensive evaluation encompassing clinical findings, radiological imaging, and sometimes biopsy. The described case highlights this approach, with X-rays and MRI revealing cortical thickening and a three-phase bone scan showing increased radiotracer uptake, indicative of an inflammatory process. A core biopsy ultimately helped rule out other differential diagnoses, emphasizing the role of histopathological examination in confirming the diagnosis. 

Treatment strategies for sclerosing osteomyelitis of Garré are not well defined, primarily focusing on symptomatic relief through analgesics, NSAIDs, and antibiotics. This case underscores the potential benefit of conservative management, with the patient achieving symptom resolution post-biopsy and continued medication. We understand that by breaking the cortex to obtain the biopsy sample, the resulting decompression helped provide relief to our patient. This is similar to what has been shown when performing core decompression in the early stages of AVN. This finding supports Nikoramov et al.’s statement regarding how a biopsy with an opening of the medullary canal sometimes decreases symptoms. However, surgical intervention remains a consideration for cases unresponsive to conservative measures.

The reported case of a 25-year-old Hispanic male with sclerosing osteomyelitis of Garré affecting the left anterior tibia midshaft is notable for several reasons. While this pathology is commonly observed in females and children [7,9], our patient was an adult male. Additionally, sclerosing osteomyelitis of Garré commonly affects the mandible [3], and our patient was affected in the tibia. Furthermore, it adds to the limited literature on the disease occurring in adult patients, particularly in long bones and among Hispanic individuals. To the best of our knowledge, only four cases of sclerosing osteomyelitis of Garré affecting the tibia of an adult have been reported [2,3,6,7], and none in the Hispanic population. The successful management of this case through biopsy and conservative treatment adds valuable insight into the therapeutic options for this rare condition.

## Conclusions

Sclerosing osteomyelitis of Garré remains a rare but significant condition, challenging clinicians with its subtle presentation and diagnostic complexities. The case presented provides a comprehensive view of its clinical manifestation, diagnostic journey, and management strategies, contributing to a broader understanding and awareness of this unique pathology. Specifically, in this case, showcasing how a biopsy can be both diagnostic and therapeutic. Further research and case reports are essential to elucidate more about its epidemiology, pathophysiology, and optimal treatment approaches, enhancing our ability to effectively diagnose and manage this rare disease. 
